# Species identification by conservation practitioners using online images: accuracy and agreement between experts

**DOI:** 10.7717/peerj.4157

**Published:** 2018-01-25

**Authors:** Gail E. Austen, Markus Bindemann, Richard A. Griffiths, David L. Roberts

**Affiliations:** 1 Durrell Institute of Conservation and Ecology, University of Kent, Canterbury, United Kingdom; 2 School of Psychology, University of Kent, Canterbury, United Kingdom

**Keywords:** Citizen science, Invasive species, Newts, Jizz, Validation, Ecological monitoring, Species observations, Crowdsourced images, Validation.

## Abstract

Emerging technologies have led to an increase in species observations being recorded via digital images. Such visual records are easily shared, and are often uploaded to online communities when help is required to identify or validate species. Although this is common practice, little is known about the accuracy of species identification from such images. Using online images of newts that are native and non-native to the UK, this study asked holders of great crested newt (*Triturus cristatus*) licences (issued by UK authorities to permit surveying for this species) to sort these images into groups, and to assign species names to those groups. All of these experts identified the native species, but agreement among these participants was low, with some being cautious in committing to definitive identifications. Individuals’ accuracy was also independent of both their experience and self-assessed ability. Furthermore, mean accuracy was not uniform across species (69–96%). These findings demonstrate the difficulty of accurate identification of newts from a single image, and that expert judgements are variable, even within the same knowledgeable community. We suggest that identification decisions should be made on multiple images and verified by more than one expert, which could improve the reliability of species data.

## Introduction

The increasing availability of new technologies has enabled those interested in the natural world to observe, identify and count species in a faster, cheaper and less intrusive manner than ever before (Pimm et al., 2015). One such use of these tools is electronic image capture from smart phones, camera traps, videos and drone footage. These images can be used to identify species (Cooper, Shirk & Zuckerberg, 2014; O’Donnell & Durso, 2014; Blaney, Pocock & Jones, 2016; Daume & Galaz, 2016; McKinley et al., 2017), and are often accompanied by informative metadata (for example, date, time and location), thus providing a wealth of information regarding species numbers, distributions and behaviours. Furthermore, these images permit identification and validation to take place at a later date, and can be shared relatively easily. A good example of this is the submission of photographs from people who wish to identify a species or validate their observation. Whether as part of a citizen science project, through a local recording group, or simply ad-hoc observations, the process involves images being uploaded electronically for identification or verification by enthusiasts of varying expertise, for example, using iSpot (http://www.ispotnature.org), iNaturalist (http://www.inaturalist.org), iRecord (http://www.brc.ac.uk/irecord/) and reddit (http://www.reddit.com/r/species) (Bates et al., 2015; Silvertown et al., 2015; Daume & Galaz, 2016; Leighton et al., 2016; Burgess et al., 2017). These online communities conduct and collaborate in species identification, but cannot always do so with certainty. For example, in iSpot contributors make a ‘Likely ID’ to remind participants that identification from images lacks certainty (Silvertown et al., 2015). Expert judgement in the identification of specimens can be sought through other means, such as wildlife trusts, local recording groups and county recorders. However, in this instance, it may be that only one person identifies or verifies the image for recording purposes. If the identification is then referred to another specialist and that person disagrees with this identification, the observation may be recorded to an agreed taxonomic level (e.g., genus). Irrespective of the route taken, expert identification and validation is widely sought.

In this context, deciding who is an expert and how expert judgements can be verified remain open questions (Goldman, 2001; Burgman et al., 2011a; Burgman et al., 2011b). Society turns to individuals with certain skills and experience for advice in decision-making (Burgman, 2015). However, this experience is often linked to qualifications or perceived ability rather than validated performance. Moreover, although expert knowledge can be generalised as information about a subject that is not universally known (Martin, Burgman & Fidler, 2012), it is often difficult to define (Hoffman, 1996). In general, expertise is domain dependent (Hoffman, 1996; Chi, 2006), dynamic (Lave & Wenger, 1991), influenced by social status (Stebbins, 1977; Ericsson, 2014), and unequally distributed within communities (Evans, 2008). Experience can make processes more automatic and reduce the effort required to complete a task, but does not necessarily lead to improved performance (Hoffman, Crandall & Shadbolt, 1998; Ericsson, 2014; Austen et al., 2016). However, defining who is an expert can be subjective and contentious, and may be perceived differently by those within a community to those outside of it (Lave & Wenger, 1991; Goldman, 2001; Ericsson, 2014; Burgman, 2015). Indeed, even within specialist communities, experts are likely to recognise certain individuals as more competent than others, and have a perception of how their own expertise compares with the rest of their peers. Ultimately, however, if an individual is perceived as an expert, they will be asked for their advice or judgement.

One area of conservation practice that relies on expert judgement is ecological monitoring (Kapos et al., 2009; Lindenmayer & Likens, 2010; Burgman et al., 2011a). Data from monitoring provide information on species numbers and distributions, including species of conservation concern, and invasive taxa that pose a threat to those species (Farnsworth et al., 2013; Latombe et al., 2017; Mang et al., 2017). One type of monitoring that incorporates both protected and invasive species is great crested newt (*Triturus cristatus*) monitoring in the UK. As a European Protected Species (EPS), *T. cristatus* is protected by law and anyone planning to survey or handle this species requires a licence. Whether professionals or volunteers, applicants for EPS licences may be expected to be familiar with all native newt species. However, instead of being required to demonstrate the relevant identification skills, applicants are required to supply a written reference from another licence holder (see Supplemental Information 3). Therefore, licences are issued according to the perceptions and opinions of other practitioners within the domain, demonstrating that both individuals, and the community as a whole, are considered to be competent in newt identification. Errors in newt identification can be costly. In England alone, the annual cost of great crested newt mitigation is estimated to be between £20 and £43 million (Lewis, Griffiths & Wilkinson, 2017). In this context, misidentification could lead to preventable delays in development, unnecessary mitigation, and potential fines for breaching the terms of a licence. This example shows the importance of accurate identification beyond reliable estimates of species numbers and distributions.

In this study, we sought to investigate the likelihood of errors in newt identification. For this purpose, we invited holders of great crested newt licences, which allows individuals to survey this species in accordance with the European Conservation of Habitats and Species Regulations 2010 and the UK Wildlife and Countryside Act 1981, to perform a simple image-sorting task. This approach is used in other research areas, such as the study of forensic human face recognition (see Jenkins et al., 2011), and provides a highly-controlled scenario for newt identification from photographs. This study design also eliminates other non-visual factors (e.g., where and when the images were taken), which removes bias associated with prior knowledge of breeding cycles and species distribution. Internet images were selected to investigate how experts sort and name images of different newt species. We also explored whether this was linked to (1) self-assessed identification ability; (2) perceived identification ability in comparison with peers; and (3) experience. In addition, we compared accuracy between professional and volunteer surveyors. Finally, we investigated whether the presence of certain diagnostic characteristics in these images was linked to increased levels of identification.

## Methods

This research was approved by the Ethics Committee of the School of Anthropology and Conservation at the University of Kent, and conducted in accordance with the ethical guidelines of the British Psychological Society.

### Participants

At the UK’s annual Herpetofauna Workers Meeting (HWM) in 2015, an invite was made for individuals in possession of a great crested newt licence to participate in a photo-sorting task. Seventeen participants (15 male, 2 female, mean age = 43 ± 13) completed the task either at the event or later at the University of Kent. All reported good or corrected-to-normal vision with spectacles or contact lenses. Informed consent was obtained from participants.

### Task design

Four species of newt found in the UK were chosen as study species, photographs of which were used as stimuli in this task. These study species comprised three native newt species, the smooth newt (*Lissotriton vulgaris*), palmate newt (*L. helveticus*) and great crested newt (*T. cristatus*), and one non-native species, the alpine newt (*Ichthyosaura alpestris*). Placed in the context of conducting an Environmental Impact Assessment (EIA), these are species that EPS licence holders are likely to come into contact with. Using the Latin binominal, photographs for each species were retrieved via the Google search engine under the ‘images’ option. Twenty unique images were chosen for each of the four species, from various websites (see Supplemental Information 2). Image selection was aimed at incorporating the range of variability that may be encountered by observers in the field. For example, selected images included males and females, newts in terrestrial and aquatic stages, and from various perspectives (i.e., dorsal, ventral, lateral and part views). The label from the downloaded image was taken as the correct species description for the purposes of this study. Although the species name assigned to the image may be incorrect, the websites of recognised organisations (e.g., conservation non-government organisations, herpetofauna fora, Wildlife Trusts, educational websites, national news outlets, etc.) took preference. The specialist nature of these sites suggests an element of validation before labelling the images, although no sources are guaranteed to be error-free. Images were randomised and numbered 1 to 80. These numbers were used to mark the reverse of the photographs in the sorting task.

### Procedure

Participants were asked to complete a short questionnaire, including age, gender and their experience with surveying amphibians in the UK (see Supplemental Information 1). This included their experience with surveying for the target species, self-perceived identification ability, self-perceived identification ability in relation to their peers, and whether they surveyed in a volunteer or professional capacity. Categories for surveying included professional, volunteering with local recording groups, organised projects, and those who survey independent of any affiliation, such as enthusiasts that monitor an area or species for personal interest. Participants performed a simple sorting task, for which they were asked to sort 80 newt images into groups according to species, irrespective of gender of the animal. Participants were supervised during the task, but no further instructions were given. Participants were also encouraged not to discuss their findings with other volunteers that had yet to participate in the task. No restrictions were placed on the number of groups created, or time taken, to avoid any undue pressure while completing the task. Once all 80 images were sorted, participants were asked to assign a species name to each group. The images were shuffled for each participant prior to the task.

### Diagnostic characteristics

To investigate whether certain aspects of these images influenced identification accuracy, each image was analysed according to the angle of view and which body parts were visible. Scores were made on perspective (namely dorsal, lateral or ventral view), the visibility of diagnostic characteristics and whether the head, head and body, or the whole newt was observable. Despite there being numerous characters defined to aid newt identification, many are linked to breeding condition, especially in males, which creates a gender and temporal bias (Arnold, Burton & Ovenden, 1978; Arnold & Ovenden, 2002). Therefore, for some defining characters their presence is only indicative for that species at certain times in the breeding cycle. For example, crests are characteristic for breeding *T. cristatus* and *L. vulgaris* males, and webbed hind feet and tail filaments are characteristic in breeding male *L. helveticus*. However, the absence of these characters does not necessarily indicate a different species, but possibly a non-breeding male or a female. Also, other characters such as colouration or ‘belly’ spots are only visible from certain angles. Furthermore, despite the ‘warty’ skin of *T. cristatus* being a defining feature, it is difficult to code for as skin will be visible on all photos, and even if the appearance is warty, it denotes whether the newt is *T. cristatus* or not, rather than differentiating *between* species. With these considerations, a binary score was limited to conspicuous hind feet and whole of the tail. Whether the animal was photographed in an aquatic or terrestrial situation may have been a factor of interest, but this could not be ascertained from every image and was therefore discounted. Inferential statistics were performed using arcsine square-root transformed proportions.

## Results

Participant experience in newt surveying averaged 13.9 years (range 4–26 years, SD = ±7.6). This was also reflected in how participants rated their identification abilities, which was either ‘very good’ (*n* = 10) or ‘good’ (*n* = 7) on a five-point scale. Moreover, most participants perceived their identification skills as ‘better than’ (*n* = 7) or the ‘same as’ (*n* = 9) their peers on another five-point scale, with only one participant considering themselves to be ‘worse than’ their peers.

In the sorting task, participants created an average of 4.7 (range 4–8, SD = ±1.1) groups of images for the four newt species. For the purposes of this study, identification was considered accurate if the species named by participants matched the species named in the downloaded image. Nine of the 17 participants correctly sorted the images into four groups and assigned the names of each of the study species to their groups. However, these groups were different for each participant, and no participant sorted their images into groups that agreed with the species named in the downloaded images (Table 1). A further seven participants assigned the names of the study species to some of their groups, but also created and named further groups. These additional groups were the Italian crested newt (*T. carnifex*) (*n* = 2), palmate/smooth newt hybrid (*n* = 2), palmate or smooth newt (*Lissotriton* spp.) (*n* = 1), and unknown (*n* = 5) (Table 1). The remaining participant created four groups, with three named as the native newts, and declared the fourth group as ‘unknown’ (Table 1).

**Table 1:** Summary of how participants sorted images into groups and the names assigned to those groups. All but one participant recognised the four study species, but eight of the participants also assigned other names or nominated the group as ‘don’t know’.

Participant	Alpine newt	Palmate newt	Smooth newt	Great crested newt	Italian crested newt	Palmate/ smooth hybrid	**Palmate*or*smooth**	**Don’t know**
1	26.3%	21.3%	27.5%	25.0%	–	–	–	–
2	25.0%	18.8%	31.3%	25.0%	–	–	–	–
3	21.3%	17.5%	31.3%	27.5%	–	–	–	2.5%
4	25.0%	16.3%	23.8%	22.5%	1.3%	3.8%	7.5%	–
5	21.3%	16.3%	26.3%	27.5%	–	–	–	8.8%
6	25.0%	28.8%	15.0%	18.8%	–	6.3%	–	6.3%
7	23.8%	20.0%	30.0%	26.3%	–	–	–	–
8	25.0%	25.0%	25.0%	25.0%	–	–	–	–
9	22.5%	21.3%	28.8%	27.5%	–	–	–	–
10	21.3%	25.0%	25.0%	28.8%	–	–	–	–
11	21.3%	25.0%	26.3%	22.5%	5.0%	–	–	–
12	23.8%	22.5%	27.5%	26.3%	–	–	–	–
13	25.0%	25.0%	25.0%	25.0%	–	–	–	–
14	25.0%	21.3%	28.8%	25.0%	–	–	–	–
15	22.5%	17.5%	32.5%	23.8%	–	–	–	3.8%
16	–	10.0%	25.0%	23.8%	–	–	–	41.3%
17	22.5%	17.5%	20.0%	25.0%	–	–	–	15.0%

Mean identification accuracy across participants was 82.7% (range 43.8–93.8%, SD = ±12.4). As participants may have recognised, but not been able to name their species groups, mean accuracy was also calculated for just the four study species (i.e., discounting unknowns and false positives). With this approach, mean accuracy increased to 87.2% (range 56.2–95.1%, SD = ±9.8) (Table 2). In addition, participants that grouped and named images as just the four study species averaged 90.6% accuracy, whereas those participants that created a ‘don’t know’ pile (*n* = 6) averaged 62.1% overall. However, mean accuracy for these participants increased to 70.1% for the study species only (Table 2). Furthermore, self-assessed ability was not an indicator of performance. For example, the highest overall accuracy score (93.8%) was achieved by three participants, two of whom believed their identification skills to be ‘very good’ and ‘better than’ their peers, and the third thought themselves to be ‘good’ and ‘same as’ their peers (Table 2). Similarly, when measuring accuracy on the study species alone, the highest accuracy was 95.1% by a participant who self-ranked as ‘good’, yet ‘worse than peers’ (Table 2).

**Table 2:** Participants’ self-assessed ability of their own identification skills, self-assessed ability compared to their peers, experience and accuracy scores in this task. Mean accuracy is reported for overall (all images) and groups named as study species. The table is ranked (descending order) by own ability, ability compared with peers, then accuracy. Neither self-assessed identification skills nor experience were indicators of accuracy.

Participant	Own ability	Ability v Peers	Mean accuracy		Experience
	(self-assessed)	(self-assessed)	Overall	Study species	(years)
1	Very good	Better than	93.8%	95.0%	20
2	Very good	Better than	93.8%	93.9%	26
3	Very good	Better than	82.5%	86.2%	25
4	Very good	Better than	81.3%	92.8%	12
5	Very good	Better than	80.0%	88.7%	17
6	Very good	Better than	78.8%	91.2%	20
7	Very good	Same as	91.3%	92.0%	6
8	Very good	Same as	90.0%	90.0%	21
9	Very good	Same as	86.3%	86.8%	12
10	Very good	Same as	85.0%	85.5%	20
11	Good	Better than	75.0%	80.3%	4
12	Good	Same as	93.8%	94.0%	11
13	Good	Same as	92.5%	92.5%	25
14	Good	Same as	88.8%	89.1%	6
15	Good	Same as	68.8%	73.1%	7
16	Good	Same as	43.8%	56.2%	7
17	Good	Worse than	81.3%	95.1%	10

**Figure 1:**
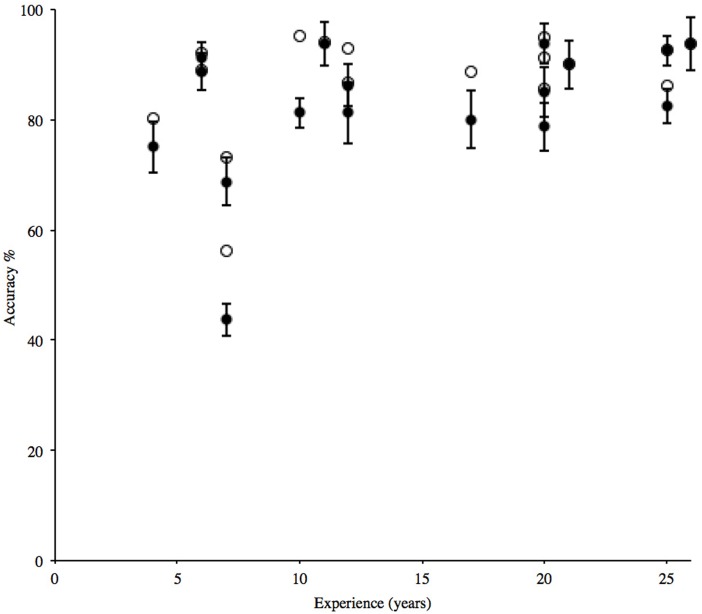
Individual percentage mean accuracy for correct solution of grouping and naming the four study species, compared with experience (filled circles). Error bars show ±1 standard error around the mean. Mean accuracy for identification of study species only (open circles) shown for comparison and follows a similar trend.

Conversely, the individual with the lowest score (43.8%) considered their identification ability as ‘good’ and ‘same as’ their peers. Their performance for just the study species was higher at 56.2%, but remained poorer than all other participants (Table 2). This is the same participant that overlooked the presence of the alpine newt in the stimuli (Table 1). A correlation of overall accuracy and number of years’ experience with newt surveying did not reach significance (*r* = 0.43, *n* = 17, *p* = 0.086), with mean accuracy on study species alone following the same trend (*r* = 0.41, *n* = 17, *p* = 0.104) (Fig. 1).

**Figure 2:**
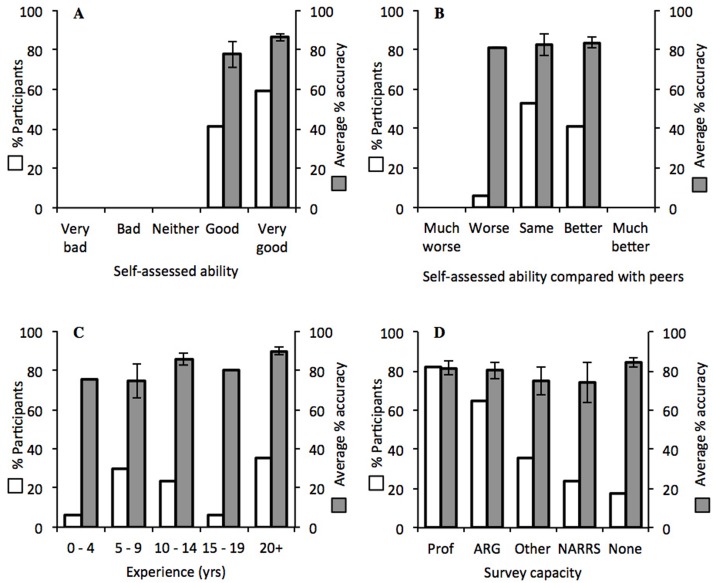
Average accuracy of participants (grey) in relation to the following factors (white): (A) self-assessed abilities; (B) self-assessed ability in comparison with peers; (C) years of experience in surveying; (D) type of surveying (“Prof”, professional, “ARG”, Amphibian and Reptile Groups, “Other”, affiliations not listed, NARRS, “National Amphibian and Reptile Recording Scheme”, and “None”, no affiliation). In (D) participants total more than 100% as 12 participants surveyed in more than one capacity. Error bars show ±1 standard error around the mean.

When comparing accuracy between participants grouped by self-assessed ability, no difference was found (*t*_15_ =  − 1.32, *p* = 0.207; Fig. 2A). In addition, a one-factor ANOVA found no difference in accuracy of participants grouped according to self-assessed ability in comparison with their peers (*F*_2,14_ = 0.03, *p* = 0.969; Fig. 2B). When analysed by experience grouped by five-year periods, average accuracy was highest for participants with experience of 20 years or more, at 89.6%, but there were no significant differences between the groups (*F*_4,12_ = 1.38, *p* = 0.297; Fig. 2C). Similarly, participants who surveyed in a professional capacity (*n* = 14) were no more accurate than those surveying as volunteers only (*n* = 3) (*t*_15_ = 0.90, *p* = 0.383; Fig. 2D).

**Figure 3:**
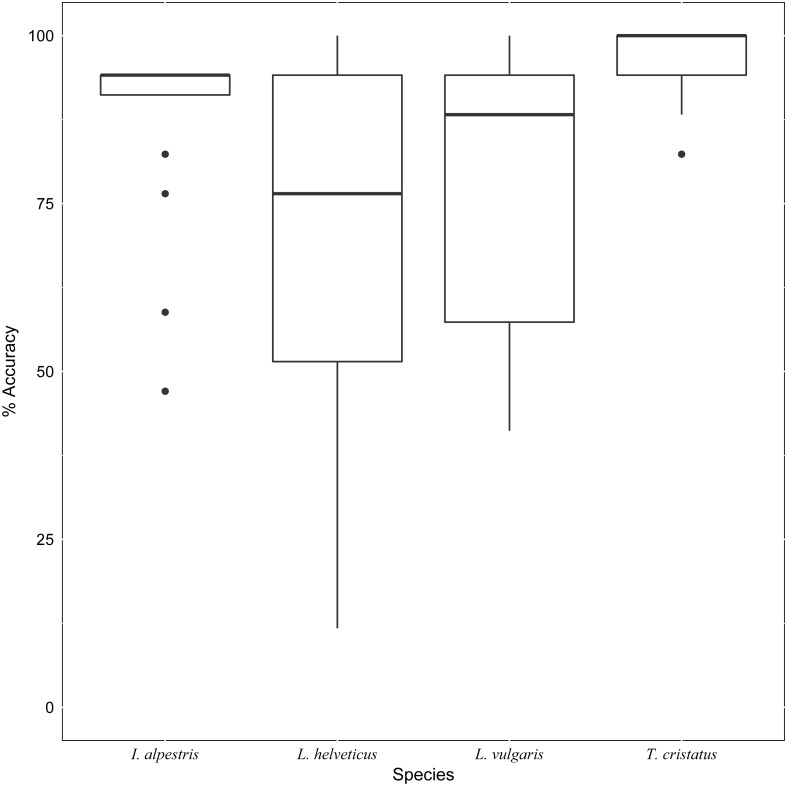
Boxplot showing accuracy rates per species. Median identification accuracy (horizontal lines in boxes) was highest for images of the target species *T. cristatus*, then *I. alpestris*, but lower for the two smaller native newts *L. helveticus* and *L. vulgaris*. The range of accurate scores was large in these two smaller newts, especially *L. helveticus*, and smaller in *T. cristatus* (with one outlier) and *I. alpestris* (with four outliers).

In addition to the differences found in individual performance, participant choice in grouping and naming images also varied according to species. For example, images of *T. cristatus* were grouped together most often, and relatively consistently (Fig. 3). Conversely, the grouping of *L. helveticus* images was highly variable (Fig. 3). A one-way ANOVA found that the consistency with which images were grouped together was highly variable between the study species (*F*_3,76_ = 7.64, *p* < 0.001). Tukey post-hoc test revealed that images of *T. cristatus* were grouped together more frequently than *L. helveticus* (*p* < 0.001) and *L. vulgaris* (*p* = 0.007). Moreover, participant agreement with the species named in the downloaded images was 95.9% for *T. cristatus*, 87.6% for *I. alpestris*, 78.5% for *L. vulgaris*, and 68.8% for *L. helveticus* images. In total, 22.5% of images were named as the same species by every participant and in agreement with the image label. There were no images for which all participants agreed on one species name, which differed from the species named in the image.

**Figure 4:**
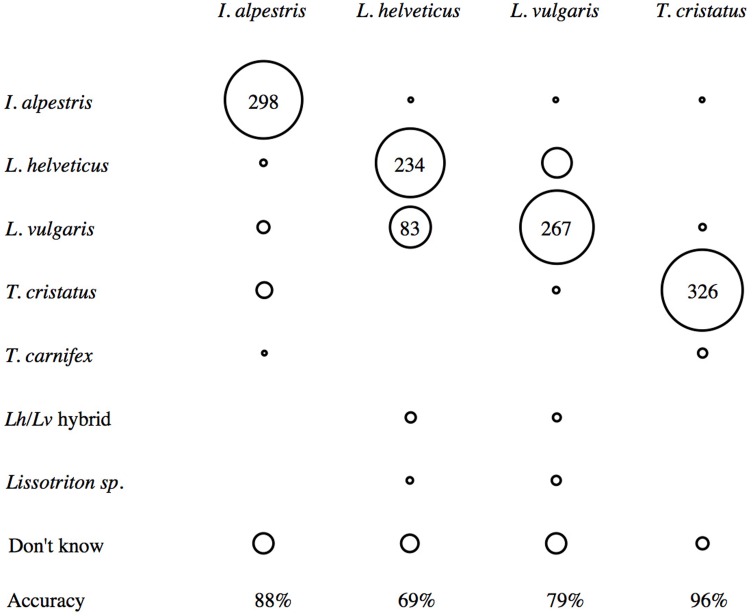
Confusion matrix showing consensus between the species name assigned to the downloaded images (columns), and how participants named images (rows). For each column (*n* = 340), percentage agreement on species name is shown at the bottom. The greatest consensus is for images of *T. cristatus*, and there is notable confusion between the two newts of the same genus, *L. vulgaris* and *L. helveticus*.

How images were grouped and named is visualised in a confusion matrix (Fig. 4). The columns are the species named in the downloaded image, and the rows are the species as named by participants. Agreement on species named in the image was highest for *T. cristatus* (95.9%), with the lack of agreement relating to participants categorising photographs as ‘unknown’ (*n* = 8), or naming images as *T. carnifex* (*n* = 4), *L. vulgaris* (*n* = 2) and *I. alpestris* (*n* = 1). Consensus in naming *I. alpestris* images was 87.6%, with the remainder being ‘don’t knows’ (*n* = 20), *T. cristatus* (*n* = 12), *L. vulgaris* (*n* = 7), *L. helveticus* (*n* = 2) and *T. carnifex* (*n* = 1). For the two newts of the same genus, the grouping of images and agreement on names was lower, with *L. vulgaris* at 79%, and *L. helveticus* at 69%. Differences here again included ‘don’t knows’ (*n* = 20 and *n* = 15 respectively), but also the concept of hybridization. Two participants created a group of images that they named as *L. helveticus/vulgaris* hybrids (these were five *L. helveticus* and three *L. vulgaris*), and one of these participants also named a group “palmate or smooth” (these were two *L. helveticus* and three *L. vulgaris*). Furthermore, misidentification between these two species was notable, with approximately 1-in-8 *L. vulgaris* named as *L. helveticus*, and nearly 1-in-4 *L. helveticus* named as *L. vulgaris* (Fig. 4). One of each species was incorrectly named as *I. alpestris*, and two *L. vulgaris* were named as *T. cristatus* (Fig. 4).

### Characteristics visible in photographs

**Figure 5:**
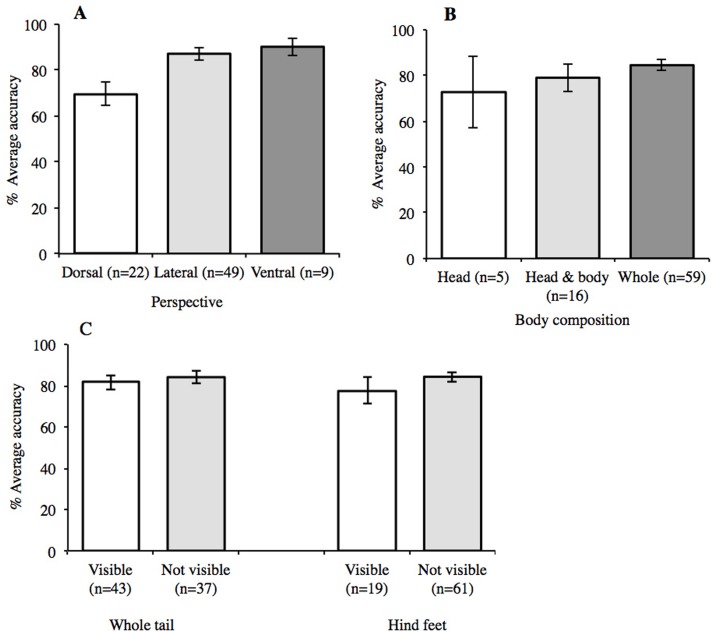
Mean accuracy relating to content and composition of images. Accuracy was highest in images showing (A) ventral perspective (90%) and (B) whole organism (85%) with no interaction between the two. Mean accuracy was not influenced by the visibility of (C) defined characteristics. Error bars show ±1 standard error around the mean.

An effect of perspective was found (Fig. 5) with a one-way ANOVA (*F*_2,77_ = 4.15, *p* = 0.019), with a post-hoc Tukey test revealing accuracy to be significantly higher in lateral than dorsal views (*p* = 0.031). Average accuracy was highest for images with ventral views (*n* = 9) (Fig. 5A). However, the behaviour of newts in the wild means that this perspective is rarely encountered unless the animal is handled. For body composition, average accuracy was highest when the whole organism was visible in the image (Fig. 5B), but a one-way ANOVA found no difference to images showing just the head, or head and body (*F*_2,77_ = 0.99, *p* = 0.377). This may seem counterintuitive, as an image of the whole organism is likely to show a greater number of features, yet one image showed just the head of *T. cristatus* and returned 100% agreement. Conversely, the lowest score (11.8%) was for an image that also showed just a head, but of the species *L. helveticus*. Moreover, 88.2% participants agreed with the identification of an image showing just the head of *L. vulgaris*, but in this image, the perspective revealed spots under the ‘chin’, which is a diagnostic feature for this species. Accuracy was not influenced by the visibility of the defined characters of hind feet or the whole tail (Fig. 5C), and no interaction was found between perspective and which parts of the body were visible.

In the 18 (22.5%) images for which *all* participants agreed on the species named in that image, 13 of these were males exhibiting breeding characteristics. These were predominantly the ‘palm’ hind feet for *L. helveticus*, and the markings, colouration and crests for *L. vulgaris* and *T. cristatus*. These temporary features relate only to males, and are easier to distinguish in water than on land (e.g., the crests fold over when out of water). The remaining five images were of female *T. cristatus*. Although they were from different perspectives, the whole body, colour, and skin texture were visible in each image.

## Discussion

This study examined how experts grouped and named images of newts downloaded from Internet sources. With a simple sorting task, participants had the opportunity to compare and contrast every image, before deciding which images belonged to the same species, and then identify those species. In this task, mean overall accuracy was 83%, with the inaccurate answers comprising ‘don’t knows’, false positives, and misidentifications of the study species. When considering correct identifications and misidentifications only (i.e., only counting images that were mistaken for one of the other study species), mean accuracy was 87%. Furthermore, judgements on species identification were inconsistent among participants, and approximately half of participants named species that were not present, or made ‘don’t know’ decisions (Table 1). Participants differed in their field experience (from four to 26 years), in their perception of their identification abilities, and the abilities of their peers, but none of these proved to be indicators of individual performance (Table 2). For example, although all participants regarded their identification ability as either ‘good’ or ‘very good’, individual overall accuracy ranged from 44% to 94%. Performance increased when only the study species were taken into account, but was still subject to broad individual differences, from 56% to 95% (Table 2). Participants were also limited in judging how their identification skills compared to that of their peers (Fig. 2).

In this task, differences between participant identification and the species named in the downloaded image were due to participants naming species not present, deciding not to assign a *species* name to a group, and confusion between the study species. As well as recognising the four study species, some participants believed images to show *T. carnifex* (*n* = 2), hybrids of the two *Lissotriton* species (*n* = 2), and only named the genus, *Lissotriton* (*n* = 1) (Table 1). In addition, six participants did not assign a species name to all of their groups (Table 1). Although approximately half of the participants (*n* = 9) grouped and named *just* the four study species, the images included in these species groups differed between participants (Table 1). However, the mean accuracy of participants naming only the four study species was 91%, compared with an average of 74% accuracy by those who designated one of their groups as ‘don’t know’. While it is possible that some participants were unable to name species, it also conceivable that they were unable to make a confident judgement from the image, and therefore refrained from doing so. This suggests limitation in accurate identification from images alone, which could be linked to image quality, image composition (Fig. 5), or even the species itself. As well as identification varying between participants, it was also highly variable among species, ranging from 96% for *T. cristatus* to 69% *L. helveticus* (Fig. 4). Even though participants agreed with the name of 23% of the images in this task, this also varied across species. Levels of agreement were found for the native newts *T. cristatus* (61%), *L. helveticus* (22%), and *L. vulgaris* (17%), but not for *I. alpestris*. Even when the images that were named as ‘unknown’ or ‘don’t know’ were removed, overall agreement was still low, at 50%.

The variability in species identification found in this study supports previous findings that agreement between experts can be inconsistent and have limitations (Burgman et al., 2011b). Participation was restricted to those in possession of an EPS licence, the issuing of which relates to competency in *field* identification rather than *image* identification. However, this restriction allowed the study of differences between *individuals*, all of whom had demonstrated a certain level of expertise within their community. While additional years of experience did not improve performance (Fig. 1), increased expertise could result in a participant being more cautious in committing to identification (Austen et al., 2016). As surveyors, participants in this study will have substantial field experience, allowing specimens to be handled and closely observed. This method of learning has been shown to be more effective than information gleaned from books alone (Culverhouse et al., 2003), and supports the concept of ‘jizz’ (see Ellis, 2011). Although the root of the word jizz is unknown, it is widely observed by field naturalists (Coward, 1922; Ellis, 2011) and taxonomists (Vane-Wright, 2000; Krell, 2004; Grove-White et al., 2007; Scharf, 2009; Williams, 2012). It corresponds with the concept of Gestalt, whereby the configuration of an object exceeds its elements and cannot be defined simply in terms of its parts (Wertheimer, 2010). In species identification, this relates to perception by which an observer can correctly name an organism without having to study its diagnostic characteristics (Ellis, 2011). For example, in this study all participants identified *T. cristatus* from an image of just a head, and this unanimous identification was probably due to the characteristic ‘warty’ skin of this species (Inns, 2009). Experience in species identification can increase the knowledge that certain features are ‘typical’ of a species, rather than being absolute, defining characteristics. For example, a recent study of mountain bongo (*Tragelaphus eurycerus isaaci*) found that the efficacy of certain morphological traits was variable, and while the inclusion of some visual traits were thought to confound accuracy, those familiar with the species made fewer misidentifications (Gibbon, Bindemann & Roberts, 2015). Some participants in the current study noted that while certain images were adequate, they were no substitute for handling an organism. Conversely, not all observers favour handling, or are permitted to handle specimens, and these restrictions can limit the observation of diagnostic characteristics. For example, this study also found that agreement on identification was higher in images presenting ventral views (Fig. 5), but this perspective is rarely experienced unless the specimen is handled.

The limitations placed on the opportunity to handle specimens may hinder accurate identification by novices. When referring to identification guides, many images are well lit with attention drawn to diagnostic features, such as secondary sexual characteristics. However, in the field newts are often observed under low light or with a torch. Furthermore, sexual dimorphism associated with breeding means that morphological differences *within* species vary throughout the year, and characteristics observable in the aquatic environment may not be visible in the terrestrial one. Of the 18 images in this task on which all participants agreed with the species name, 13 were of males in breeding condition. Such variation may confuse novice observers, but the rise of citizen science and availability of technology means that expert judgement can be sought via images. In these instances, guidance on what is useful to include in a photograph may help the identification process. Participants in this study were asked to complete the task on an individual basis to help avoid biases that can arise in groups of interacting experts (McBride et al., 2012). However, combined judgements can have an advantage over decisions by individuals (Surowiecki, 2004; White et al., 2013; Swanson et al., 2016). While our findings support seeking multiple opinions where possible, this may not be achievable or practical in all situations. Nevertheless, even with numerous experts making decisions on the same images without time limits, participants in this task agreed on the species name for less than one-in-four of all images, demonstrating the difficulty of naming an organism from a single image.

This study also highlights some of the issues associated with testing identification accuracy and expert participation experimentally. By definition, expert knowledge is knowledge on a subject not commonly known (Martin, Burgman & Fidler, 2012). Consequently, experts form only a small part of the general population. In addition, not all experts may be willing to undergo experimental assessments of their ability, thus limiting expert participant pools further. Selecting stimuli can also be problematic, as most have the potential to contain some element of error. For example, a study by Culverhouse et al. (2003) found that of specimens that had been labelled by the author and validated by an independent taxonomist, expert consensus on specimen names was just 43%. Similarly, the current study found expert agreement with species named in downloaded images to be highly variable, even though images were sourced from websites dedicated to species identification and herpetofauna (e.g., iSpot, iNaturalist, Amphibian and Reptile Groups, and Amphibian and Reptile Conservation Trust). Regardless of the source, the extent to which the reliability of an online identification can be ascertained is linked to the perceived expertise of a person (Eiser et al., 2009). Even though participants’ expertise is linked to identification in the field rather than from photographs, many are approached for image identification or validation. Although the use of live specimens may have been a better indicator of individual identification abilities, this would be impractical for testing different participants with the same stimuli. Identification and consensus in this task may have also been improved if the stimuli images had been presented under optimised conditions (e.g., images taken from identification guides). However, analogous research on forensic face matching suggests that experiments controlled in this manner underestimate errors, as they do not account for the natural variation found in realistic settings (Megreya, Sandford & Burton, 2013). While image identification differs from field identification, this study reveals variation in judgement for the same stimuli, within an expert group. Moreover, although the number of species in this study was limited to four, expert judgement in this constrained context was variable. Decisions required on more speciose groups could create greater levels of disagreement.

In summary, the results in this study suggest that consensus may be a more appropriate indicator than accuracy in species identification. Apart from a handful of well-known species, accurate identification often requires the skills of taxonomic specialists (Pimm et al., 2015). However, we found that when presented with the same stimuli, the grouping and naming of images was inconsistent across participants (Table 1) and species (Fig. 3). While there are philosophical debates around trusting experts and their decisions (Goldman, 2001; Burgman, 2015), expert judgements remain crucial for ecological monitoring, especially at a time where improvements in technology are creating volumes of images. While our research asked field experts to make judgements on images, this study concurs with other studies in finding that consistent identification is not linked to experience, or that expertise is the domain of the professional (Burton et al., 1999; White et al., 2014; Landrum & Mills, 2015; Austen et al., 2016). Although there are few studies directly comparing identification accuracy between experts, the available evidence also reveals variable performance (e.g., Culverhouse et al., 2003).

Given the heterogeneous nature of species variation, a repeat of this study with different taxa would discover if identification rates are comparable. Moreover, as most participants in this study carried out surveys in more than one capacity, a repeat with contributors that are just professionals *or* volunteers could build on our findings. Finally, although images from novices can provide useful information in a timely manner (Silvertown et al., 2015; Daume & Galaz, 2016), they can present challenges to those asked to identify those images. While data from large volumes of digital images can be novel, expedient and revealing, they are not necessarily as informative as the indefinable ‘jizz’ gained from field observations.

##  Supplemental Information

10.7717/peerj.4157/supp-1 Supplemental Information S1 Participant questionnaire

10.7717/peerj.4157/supp-2 Supplemental Information S2 Links to online image sources A list of website links for each of the images used in this study (downloaded August 2014).

10.7717/peerj.4157/supp-3 Supplemental Information S3 Natural England EPS reference form Natural England form used to provide reference in support of an application for a European Protected Species licence.

## Additional Information and Declarations

### Data Availability

The following information was supplied regarding data availability: University of Kent data repository: http://kar.kent.ac.uk/65753/.
