# Supplementary material for: Species identification by conservation practitioners using online images: accuracy and agreement between experts
Source: PeerJ. 2018 Jan 25;6:e4157. doi: 10.7717/peerj.4157 (PMC5787348; doi:10.7717/peerj.4157)
Supplement: Supplemental Information S1 [file peerj-06-4157-s001.docx]

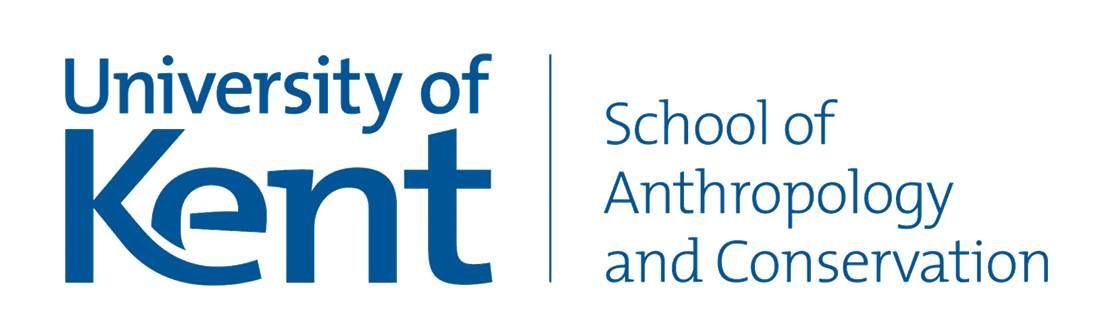


**S1. Participant questionnaire**

Participant___________

Many thanks helping with this survey. The survey is anonymous, but we would like to gather some information about you.

Do you agree to participate in this survey? Yes No

1. What is your gender?

Female Male Other

2. Which age range applies to you?

under 18 18-24 25-34 35-44

45-54 55-64 65+

3. Do you consider yourself to have normal vision?

Yes No, but my vision is corrected to normal with glasses or contact lenses

No, I have a visual impairment

4. Do you hold a great crested newt licence?

Yes When did you obtain your first gcn licence?

No

5. How do you rate your ability to identifying amphibians found in the UK?

Very bad Bad Neither good nor bad Good Very good

6.Have you been involved in survey work that involves newts in the last 5 years?

Yes No

7. If yes, is it in any of the following capacities (please tick all that apply):

Professional With an ARG NARRS

No affiliation Other:

8. If you have surveyed in a non-professional capacity, in which area(s)?

(*E.g. Kent, Canterbury, CT1)* _______________________________________________

9. Are you involved with training other people?

Yes No

10. How do you rate your ability identifying amphibians in comparison to your peers?

Much worse Worse Same as peers Better Much better

Thank you very much for answering these questions.

Please now continue to the image matching task.
