# Supplementary material for: Species identification by conservation practitioners using online images: accuracy and agreement between experts
Source: PeerJ. 2018 Jan 25;6:e4157. doi: 10.7717/peerj.4157 (PMC5787348; doi:10.7717/peerj.4157)
Supplement: Supplemental Information S2 — A list of website links for each of the images used in this study (downloaded August 2014). [file peerj-06-4157-s002.docx]

**Supplementary Information**

**S2. A list of website links to image sources (downloaded August 2014)**

***Ichthyosaurus alpestris***

<http://www.club100.net/species/M_alpestris/M_alpestris.html>

<http://www.thinkreptiles.com/amphibians/salamander-species-index>

<http://www.herpetofauna.co.uk/alpine_newt.htm>

<http://www.telegraph.co.uk/women/sex/valentines-day/9079601/Animal-love-Valentines-day-lovebirds-in-displays-of-affection.html?image=13>

<http://www.amphibian.co.uk/alpine.html>

<http://www.museumkiev.org/zoo/cadastre/triturus_alpestris_en.html>

<http://www.herp.it/indexjs.htm?SpeciesPages/TrituAlpes.htm>

<http://www.kentarg.org/alpine-newt-mesotriton-alpestric-formally-triturus-alpestris.html>

<http://www.herpetofauna.co.uk/alpine_newt.htm>

<http://www.freenatureimages.eu/animals/Amphibia,%20Amfibieen,%20Amphibians/Triturus%20alpestris,%20Alpine%20Newt/index.html#Triturus%20alpestris%2020%2C%20male%2C%20Alpenwatersalamander%2C%20Saxifraga-Willem%20van%20Kruijsbergen.jpg>

<http://www.schule-bw.de/unterricht/faecher/biologie/projekt/amphibien/ichthyosaura_alpestris.html>

http://www.arc-trust.org/non-native-amphibians.html

<http://www.planetepassion.eu/amphibians-in-france/Alpine-Newt-France.html>

<http://www.caudata.org/cc/species/Triturus/T_alpestris.shtml>

<http://www.iucnredlist.org/details/59472/0>

<http://www.caudata.org/forum/f1173-advanced-newt-salamander-topics/f24-photo-gallery-video-gallery-technique-discussion/f25-photo-video-gallery/64473-mesotriton-alpestris-apuanus.html>

<http://www.surrey-arg.org.uk/SARG/08000-TheAnimals/SARG2Amphibians.php>

<http://www.nahuby.sk/obrazok_detail.php?obrazok_id=69312>

<http://forum.serpenti.it/viewtopic.php?f=83&t=16224>

<http://www.ufz.de/index.php?en=16938>

***Lissotriton helveticus***

<http://www.caudata.org/cc/species/Triturus/T_helveticus.shtml>

<http://www.caudata.org/cc/species/Triturus/T_helveticus.shtml>

<http://inpn.mnhn.fr/espece/cd_nom/444432>

<http://www.surrey-arg.org.uk/SARG/08000-TheAnimals/SARG2Amphibians.php>

<http://www.herpetofauna.co.uk/palmate_newt.asp>

<http://www.devonwildlifetrust.org/species/palmate-newt>

<http://www.butemuseum.org.uk/natural-history-gallery/fauna/reptiles-and-amphibians/>

<http://www.arkive.org/palmate-newt/triturus-helveticus/image-A7572.html>

<http://www.arkive.org/palmate-newt/triturus-helveticus/image-A7570.html>

<http://www.arkive.org/palmate-newt/triturus-helveticus/image-A6802.html>

<http://www.arkive.org/palmate-newt/triturus-helveticus/>

<http://www.kentarg.org/Amphibians/palmate-newt>

<http://www.kentarg.org/Amphibians/palmate-newt>

<http://www.naturespot.org.uk/species/palmate-newt-1>

<http://www.devonwildlifetrust.org/species/Palmate+newt/>

<http://www.bbc.co.uk/nature/22120791>

<http://www.uk-wildlife.co.uk/palmate-newt-lissotriton-helveticus-male/>

https://www.google.co.uk/search?q=lissotriton+helveticus&client=firefox-a&hs=nFT&rls=org.mozilla:en-US:official&channel=sb&source=lnms&tbm=isch&sa=X&ei=SOxsU62jDYrYPL3ZgLAL&ved=0CAYQ_AUoAQ&biw=900&bih=817#facrc=_&imgdii=_&imgrc=rPjKSXddI3EznM%253A%3BgalN_OqrOSoe1M%3Bhttp%253A%252F%252Fiucnredlist-photos.s3.amazonaws.com%252Fmedium%252F1159557300.jpg%253FAWSAccessKeyId%253DAKIAJIJQNN2N2SMHLZJA%2526Expires%253D1430413300%2526Signature%253DKhh5XwPEE2cFL%25252FPIZ3OF6UJEo%25252B4%25253D%3Bhttp%253A%252F%252Fwww.iucnredlist.org%252Fdetails%252F59475%252F0%3B480%3B320

http://www.arc-trust.org/palmate-newt.html

http://www.arc-trust.org/palmate-newt.html

***Lissotriton helveticus***

<http://www.arkive.org/smooth-newt/triturus-vulgaris/image-A9678.html>

<http://carlcorbidgefieldherping.blogspot.co.uk/2011/08/evidence-of-successful-reptilian.html>

<http://surrey-arg.org.uk/SARG/08000-TheAnimals/SARG2Amphibians.php>

<http://www.euroherp.com/species/Lissotriton_vulgaris/>

<http://www.wildlifetrusts.org/species/smooth-newt>

<http://www.inaturalist.org/check_lists/15625-Karachay-Cherkess-Check-List>

<http://www.wildaboutbritain.co.uk/gallery/files/2/8/7/4/herplvmalehome02a.jpg>

<http://www.naturespot.org.uk/species/smooth-newt-0>

<http://www.naturalis-historia.de/cgi-bin/Seite.pl?Systematisch;Animalia;Amphibia%20-%20Lurche;3#titel>

<http://www.arkive.org/smooth-newt/triturus-vulgaris/image-A8789.html>

<http://www.arkive.org/smooth-newt/triturus-vulgaris/image-A22254.html>

<http://www.kentarg.org/Amphibians/smooth-newt>

<http://www.froglife.org/amphibians-and-reptiles/smooth-newt/>

<http://www.froglife.org/amphibians-and-reptiles/smooth-newt/>

<http://www.bto.org/volunteer-surveys/gbw/gardens-wildlife/garden-reptiles-amphibians/a-z-reptiles-amphibians/smooth-newt>

http://www.froglife.org/amphibians-and-reptiles/smooth-newt/

<http://www.nederlandsesoorten.nl/nsr/concept/0AHGPYIHYLLN/biology>

<http://froglife-frogbites.blogspot.co.uk/2012/03/froglifes-dragon-of-month-smooth-newt.html>

<http://www.arkive.org/smooth-newt/triturus-vulgaris/image-A10523.html>

<http://www.biolib.cz/en/taxonimage/id37329/>

***Triturus cristatus***

<http://www.arkive.org/great-crested-newt/triturus-cristatus/>

<http://commons.wikimedia.org/wiki/File:Triturus_cristatus_Sinarp_8.JPG>

<http://www.oxfordshire-arg.org.uk/great_crested_newt1.htm>

<http://www.biolib.cz/en/image/id10824/>

<http://www.kentarg.org/Amphibians/great-crested-newt>

<http://www.kentarg.org/Amphibians/great-crested-newt>

<http://news.bbc.co.uk/local/gloucestershire/hi/people_and_places/nature/newsid_8314000/8314899.stm>

<http://www.arkive.org/great-crested-newt/triturus-cristatus/image-A22844.html>

<http://www.theguardian.com/environment/2012/apr/01/specieswatch-newt-ponds-conservation>

<http://www.arkive.org/great-crested-newt/triturus-cristatus/image-A22870.html>

<http://www.herpetofauna.co.uk/great_crested_newt.asp>

<http://calphotos.berkeley.edu/cgi/img_query?enlarge=0000+0000+0610+1121>

<http://calphotos.berkeley.edu/cgi/img_query?enlarge=0000+0000+1210+2741>

<http://biodiversitatecbc-apmis.ro/new/?page=galerie>

<http://www.iucnredlist.org/details/22212/0>

<http://www.bbcwildlife.org.uk/node/3061>

<http://www.arkive.org/great-crested-newt/triturus-cristatus/image-A18992.html>

<http://www.euroherp.com/Resources/Trips/up/128-1361207113.jpg>

<http://speciesofuk.blogspot.co.uk/2013/07/week-21-great-crested-newt-triturus.html>

<http://www.biolib.cz/en/taxonimage/id10829/?taxonid=309>
