# Supplementary material for: Species identification by conservation practitioners using online images: accuracy and agreement between experts
Source: PeerJ. 2018 Jan 25;6:e4157. doi: 10.7717/peerj.4157 (PMC5787348; doi:10.7717/peerj.4157)
Supplement: Supplemental Information S3 — Natural England form used to provide reference in support of an application for a European Protected Species licence. [file peerj-06-4157-s003.docx]

**Wildlife Management and Licensing**

**Reference in support of licence applicant**

References provided in support of a licence application consist of two elements:

- a statement from the referee, demonstrating their own skills, relevant experience and qualifications to provide this reference; and
- a statement by the referee on the skills and experience of the applicant for this licence.

See: <https://www.gov.uk/government/publications/reference-to-support-a-protected-species-licence>

for guidance on providing references.

| **1. Summary of licence sought** | |
| --- | --- |
| Species to be affected | Techniques to be licensed |

|  |  |
| --- | --- |
|  |  |
|  |  |
|  |  |
|  |  |

To add more rows to this table, click inside the table, then click <<Table, Insert, Rows Below>>

| **2. Referee Contact Details** *(We may contact you to discuss this reference.)* | | | | | | |
| --- | --- | --- | --- | --- | --- | --- |
| **Name** | Title | Forename | | | Surname |  |
|  |  |  | | |  |  |
| **Address** |  | | | | |  |
| **Postcode** |  | | **Email** |  | |  |
| **Telephone** |  | | **Mobile** |  | |  |

| **3. Referee’s Skills and Experience**  Please provide concise details of your relevant skills and experience, which enable you to comment in support of this application. You should provide reference numbers for any licences held in the last 3 years for similar species and techniques. |
| --- |

|  |
| --- |

| **Previous Licence Ref Numbers** |  |
| --- | --- |

| **4. Referee’s Statement on the Applicant’s Skills and Experience**  The referee should provide details of **their knowledge** of the **applicant’s experience and skills** in working with the species (or similar species) and techniques listed in Section 1 above. Please refer to relevant licences under which the applicant has acted as an agent or assistant and any relevant training undergone by the applicant. | |
| --- | --- |
| **Applicant Name** |  |
| Species worked on: | |

|  |
| --- |

| Activities and Methods (Techniques) used: |
| --- |

|  |
| --- |

| Please state how long you have known/worked with the applicant |  |
| --- | --- |
| For applications relating to specific time-limited projects, please tick here to confirm that you are aware of the scope/scale of the project to be carried out under this licence. |  |

| **Referee Signature** *(e-signature acceptable)* | **Date** |
| --- | --- |

|  |  |
| --- | --- |

*For references submitted via email, please insert an electronic signature above or tick this box to confirm the details you have provided are a true and accurate record*
